# Sex Differences in Epidemiology, Morphology, Mechanisms, and Treatment of Mitral Valve Regurgitation

**DOI:** 10.3390/medicina59061017

**Published:** 2023-05-24

**Authors:** Gregorio Tersalvi, Lorenzo Gaiero, Michele Capriolo, Yvonne Cristoforetti, Stefano Salizzoni, Gaetano Senatore, Giovanni Pedrazzini, Luigi Biasco

**Affiliations:** 1 Department of Cardiology, Cardiocentro Ticino Institute, 6900 Lugano, Switzerland; 2 Department of Internal Medicine, Ente Ospedaliero Cantonale, 6850 Mendrisio, Switzerland; 3 Division of Cardiology, Azienda Sanitaria Locale Torino 4, Ospedale di Ciriè, 10073 Ciriè, Italy; 4 Division of Cardiology, Ospedale Gradenigo, Humanitas Torino, 10153 Turin, Italy; 5 Division of Cardiac Surgery, Città della Salute e della Scienza, Università degli Studi di Torino, 10126 Turin, Italy; 6 Faculty of Biomedical Sciences, Università della Svizzera Italiana (USI), 6900 Lugano, Switzerland

**Keywords:** mitral regurgitation, sex differences, transcatheter mitral valve repair

## Abstract

Sex-related disparities have been recognized in incidence, pathological findings, pathophysiological mechanisms, and diagnostic pathways of non-rheumatic mitral regurgitation. Furthermore, access to treatments and outcomes for surgical and interventional therapies among women and men appears to be different. Despite this, current European and US guidelines have identified common diagnostic and therapeutic pathways that do not consider patient sex in decision-making. The aim of this review is to summarize the current evidence on sex-related differences in non-rheumatic mitral regurgitation, particularly regarding incidence, imaging modalities, surgical-derived evidence, and outcomes of transcatheter edge-to-edge repair, with the goal of informing clinicians about sex-specific challenges to consider when making treatment decisions for patients with mitral regurgitation.

## 1. Introduction

Non-rheumatic mitral valve regurgitation (MR) is one of the predominant valvular heart diseases in western countries, with an incidence that increases with age [1].

Several sex-related disparities have been recognized in the incidence, pathological findings, and pathophysiological mechanisms of MR, as well as in diagnostic accuracy of currently available imaging tools. In addition, the likelihood of access to treatments and outcomes for both surgical and interventional therapies among women and men with MR appears to be dissimilar [2].

Nonetheless, current European and US guidelines identified common diagnostic and therapeutic pathways encompassing medical, surgical, and transcatheter treatments for both women and men that do not take into account patient sex as factor impacting on the decision-making process [3,4].

This review aims at summarizing current knowledge on sex-related differences in incidence, imaging modalities, surgical derived evidence, and outcomes of transcatheter edge-to-edge mitral valve repair (TEER) to inform clinicians about sex-specific challenges to consider when making treatment decisions for patients with MR.

## 2. Epidemiology

Mitral regurgitation (MR) is the most common valvular heart disease, affecting about 1% to 2% of the world’s population with a prevalence that increases to 7% to 9% among patients over 75 years of age [1]. Prevalence in the western countries is thought to be greater because of higher life expectancy. In the Framingham Heart Study, the reported prevalence of MR, defined as more than mild, was 19.0% in men and 19.1% in women [5]. In a cohort study reviewing 6851 consecutive individuals without suspected valve disease, the overall prevalence of more than moderate MR was 11.7% in male and 12.5% in female patients [6].

Primary (or degenerative) MR is usually caused by anatomical abnormalities of mitral leaflets, such as mitral valve prolapse (MVP), whereas secondary (or functional) MR results from abnormal left ventricular (LV) size, shape, or function, mainly in the context of heart failure [7]. The evaluation, treatment, and prognosis in patients with these conditions do differ significantly. Moreover, sex is associated with different distribution of MR aetiologies, with males more affected by the secondary form, in particular when associated with ischemic heart disease, and women more often affected by primary MR.

In a large European prospective cohort reviewing 63,463 echocardiographic studies performed for any indication, significant MR was present in up to 5% of all studies [8]. Among patients with MR, sexes were similarly represented (male 53%, female 47%). However, women were, on average, 3.1 years older than men. Severe LV dysfunction, defined as LV ejection fraction (LVEF) < 30%, was significantly more common in men, whereas severe calcification of the mitral apparatus was more common in women [8].

Among causes of primary MR, MVP affects 2% to 3% of the population and occurs more commonly in women, but men predominate in patients undergoing mitral valve surgery or intervention [9,10,11,12,13,14]. In a large cohort study from the Mayo Clinic assessing 8229 patients with MVP [15], women with severe MR were less likely than men with severe MR to undergo mitral valve surgery (52% vs. 60%, adjusted risk ratio, 0.79 [95% CI, 0.74 to 0.84]). At 15 years, women with no or mild MR had better odds of survival than men (87% vs. 77%, adjusted risk ratio, 0.82 [CI, 0.76 to 0.89]), but those with severe regurgitation had worse survival than men (60% vs. 68%, adjusted risk ratio, 1.13 [CI, 1.01 to 1.26]). The survival rate 10 years after surgery was similar in women and men [15].

Mitral annular disjunction (MAD) is associated with MVP and has been shown to be a risk factor for ventricular arrhythmias [16]. MAD is defined as an abnormality of the mitral annulus, in which the posterior mitral leaflet inserts into the left atrial wall, separate from the LV myocardium, which is normally in contact with the mitral annulus [17]. Several factors may be associated with a higher MAD frequency in patients with MVP. Putnam et al. retrospectively investigated 90 patients with severe MVP and MR who underwent preoperative computed tomography. MAD was detected in 20% of these patients, being most often associated with female sex, smaller mitral annulus size, and greater posterior leaflet length [17]. Furthermore, the malignant bileaflet MVP syndrome, described in previous literature, is characterized by bileaflet MVP, frequent ventricular ectopy, and female sex [18,19].

Opposite to primary MR, secondary MR is more frequent in men than women [8,13,20]. This is because secondary MR develops in the setting of LV dysfunction, i.e., heart failure with reduced ejection fraction, which has higher prevalence among males [21,22]. Ischaemic LV dysfunction is significantly more common in men, whereas non-ischaemic aetiology is more prevalent in women [20]. However, women seem at disproportionately elevated risk of developing MR after myocardial infarction or in the context of coronary artery disease [23]. Regarding comorbidities and risk factors, women referred for mitral valve surgery for secondary MR more often have comorbid hypertension and less often have ventricular arrythmias or a history of smoking, compared with men [24]. Furthermore, women have less dilated ventricles and less severe MR but may have worse heart failure symptoms and worse mental health scores [24]. Sex differences were observed also in the treatment strategies, where women with secondary MR more often underwent surgical mitral valve replacement when compared with men, although no differences were observed for TEER [20].

## 3. Morphology and Imaging

### 3.1. Sex-Specific MR Phenotypes

Compared to male patients, female patients have different anatomical, morphological, and pathophysiological features [25].

First, women have smaller atrial and ventricular dimensions as well as smaller regurgitant volumes. These differences persist even after indexing the measurements for body size [15,26]. In the setting of primary MR, MVP is the most prevalent cause in western countries and in the female population [1,10,25]. It encompasses a broad spectrum of phenotypes that significantly impact on technical aspects and outcomes of any type of intervention, both surgical and percutaneous. In this setting, women present less frequently with flail leaflets or posterior leaflet prolapse and more often with thicker and myxomatous valves with multiscallop and anterior prolapse, thus challenging both surgical correction as well as TEER [15]. A distinct sex-related remodeling in the extracellular matrix can explain the morphological presentation and the different prevalence of complex lesions in females leading to a higher procedural complexity of surgical repair [2].

Furthermore, mitral annulus calcification is more frequently encountered in women and, irrespective of the degree of its extension, it is a predictor of mortality and mitral valve replacement in surgical patients [27].

Finally, functional atrial MR (AMR) has been recently recognized as a new pathophysiological entity. AMR is defined as leaflet malcoaptation caused by isolated annular dilatation with insufficient leaflet growth and impaired annular dynamics in patients with atrial fibrillation and/or heart failure with preserved ejection fraction [28]. This mechanism of MR has a high incidence and significant prognostic implication. Studies have shown a greater frequency of AMR in women [28,29]. Several factors might explain this association such as increased atrial fibrosis, higher levels of inflammation markers, differences in the histological composition of the annulus with reduced elastic components, and less myocardium leading to a higher degree of dilatation [29].

**Figure 1:**
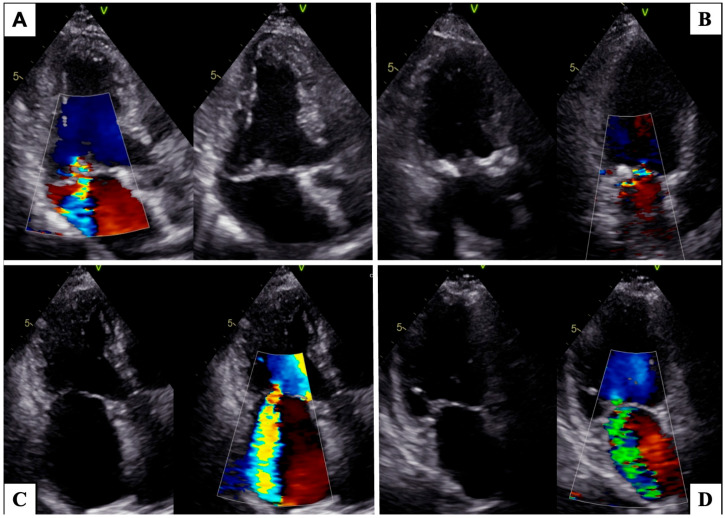
Morphological features more frequently found in female patients on bidimensional echocardiography. Barlow’s disease with multi-scallop and bileaflet prolapse (**A**). Extensive mitral annular calcification (**B**). Excessive annular dilatation in atrial functional mitral regurgitation (**C**). Posterior leaflet tethering in ischemic functional mitral regurgitation (**D**).

Figure 1 shows morphological features frequently encountered in female patients with MR.

### 3.2. Multimodality Imaging in MR Assessment

Two-dimensional and three-dimensional echocardiography represent the cornerstone of MR quantification. Even if several studies support current guidelines for quantitative and qualitative MR quantification and indications for mitral valve intervention, many pitfalls that can delay referrals in women must be considered. Indeed, current guidelines do not include sex-specific criteria to adequately account for sex-related differences, do not mandate indexing volumes, and their evidence is based on studies derived from populations with male predominance [3,4,30,31]. Although it is clearly established that women have smaller cardiac chambers and effective regurgitant orifice area (EROA), even when indexed for smaller body surface area (BSA), a lower percentage of women reach current definition of severe MR and for mitral valve intervention [15,26]. As a consequence, women referred for mitral valve surgery in degenerative MR had larger LA and LV volumes after indexing for BSA. Moreover, women with functional MR referred for TEER have smaller annular area, distance between the papillary muscles, and EROA, while their EROA/left ventricular end-diastolic volume ratio is larger, thus suggesting more severe degrees of regurgitation [32,33]. So, especially in women, if doubts persist about correct quantification of MR, or significant discrepancies are evident between reported symptoms and echocardiographic findings, several other tools are available to guide the decision-making process.

The assessment of vena contracta area with 3D echocardiography allows direct measurement of EROA, eliminating geometric and flow assumption of 2D echocardiography. Compared to 2D PISA methods, which are based on the limiting supposition that EROA is circular, it improves the accuracy of MR grading and is highly sensitive for definition of severe primary MR [34,35].

In current guidelines, reduction in LVEF is a main parameter for mitral valve surgery [3,4]. However, this parameter has significant intraobserver and interobserver variability and detects myocardial disfunction later in the disease course. Left ventricular global longitudinal strain (LV-GLS) is a reproducible, accurate, and load-independent echocardiographic parameter that can detect early subclinical LV dysfunction in asymptomatic patients [35]. Speckle tracking echocardiography permits assessment of myocardial deformation before LVEF declines, and it has shown significant prognostic value to predict adverse clinical outcomes in patients with LVEF > 60% undergoing mitral valve repair [36,37]. Therefore, LV-GLS may provide additional prognostic value in patients with asymptomatic or undefined MR, and it has been proposed as a possible parameter for referring to cardiac surgery. The main limitations of this technique are that image post-processing is time-consuming, that optimization of image acquisition is imperative to ensure adequate image quality, and that inter-vendor variability makes standardized GLS application challenging.

However, the most remarkable results in MR quantification have been achieved by cardiac magnetic resonance imaging (CMR). CMR is the gold standard to assess chamber volume quantification and thus to evaluate LV and left atrial dilatation, and it is also the most accurate method for quantification of regurgitant volumes. The use of CMR reclassifies a substantial number of patients with MR defined as severe by echocardiography [38]. Furthermore, CMR is an independent predictor of LV reverse remodeling after MR correction whereas echocardiography is not [39]. Therefore, most recent studies suggest that CMR-specific thresholds may be more appropriate and more closely related to outcome compared to echocardiography, and CMR seems to be the most reliable method to identify patients with severe MR that will benefit most from the intervention.

Finally, natriuretic peptides are the most widely used markers of cardiac volume overload in daily clinical practice. Their application in the assessment of patients with valvular heart disease is emerging, and many studies have shown a strong correlation between natriuretic peptides and clinical outcomes in patients with symptomatic and asymptomatic MR [40,41]. Although specific cut-offs have not yet been defined, they can be another useful tool for implementing the management of patients with MR when indication for intervention is not clear. Importantly, sex is one of the factors together with BMI and age that influence the values of natriuretic peptides. However, the performance of these peptides for diagnosing heart failure and their prognostic utility are similar in both sexes, and sex-specific cut-off points are not usually recommended [42,43,44].

Therefore, quantification of MR, particularly in the decision-making process driving timing of intervention, should not only be focused on conventional echocardiographic parameters but must include, with a holistic approach, different diagnostic tools and patients features, especially in women.

## 4. Surgical Therapy

Sex differences have been described in both mitral valve pathology, surgical approaches, and clinical outcomes after mitral valve surgery.

From an anatomical perspective, in relation to their smaller body surface area, women show smaller LV dimensions and, thus, smaller mitral valve annular dimensions, as compared with men [15].

From a pathological perspective, diffuse calcifications of the mitral annulus and leaflets are commonly encountered in females, while isolated prolapse of the posterior leaflet is more common in males [15]. Furthermore, the incidence of mitral stenosis, as a standalone pathology or in association with MR, is higher in females [45].

These pathological characteristics translate into different surgical approaches between males and females as well as relevant discrepancies in outcomes.

Firstly, women are less likely to be referred for surgical interventions as compared to men, raising the issue of a large unmet need for treatment in females [15]. Furthermore, referral delays cause women to undergo surgical mitral valve intervention at an older age as compared to their male counterparts along with an over-representation of women in non-elective patients, thus increasing their procedural risk [46]. In addition, women are often referred to surgery at a later disease stage with higher New York Heart Association (NYHA) class at presentation. Finally, because of the greater presence of diffuse calcifications of the mitral leaflets, mitral valve repair represents a challenging procedure. Thus, females are more frequently treated with mitral valve replacement as compared to men, with an obvious impact on long-term outcomes.

Female patients undergoing mitral valve surgery have, as well, a higher operative mortality and lower long-term survival as compared to males. This finding is only partially explained by the presence of associated comorbidities [46]. In the setting of primary MR, mitral valve repair was able to restore life expectancy in men. Nonetheless, this prominent prognostic achievement was not mirrored in women that showed the persistence of a relevant excess in mortality after surgical repair [46].

Female sex is also included as a covariable in surgical risk prediction tools such as the EuroSCORE II and the Society of Thoracic Surgeon score (STS score), given its independent association with adverse outcomes after surgery [47].

In summary, surgically derived data show that females are less likely referred to MR surgical correction, often scheduled at a later disease stage. Their anatomical characteristics do often impact on the choice between repair and replacement with a lower likelihood of mitral valve repair. In addition, even when adequately repaired, their life expectancy is not fully restored as is in their male counterparts. Reasons for these discrepancies are only partially explained so far and might depend upon associated comorbidities, as well as with an intrinsic risk derived from female sex.

## 5. Interventional Therapy

### 5.1. Evidence from Randomized Clinical Trials

The first comparative trial assessing safety and clinical efficacy of TEER versus surgery was the EVEREST II trial [48]. The primary endpoint was set as the composite of freedom from death, mitral valve surgery, and recurrent MR grade ≥3 at 12 months. The study enrolled highly selected patients fulfilling strict echocardiographic criteria. This led to the inclusion of patients with predominant degenerative MR (73%) while females were almost one third in both study groups (38% TEER, 34% Surgery). Sex interaction was analyzed in a pre-specified post-hoc subgroup. Among female patients, freedom from death, surgery, and recurrent MR was observed in 37 (55%) out of 67 patients enrolled in the TEER arm as compared to 22 (73%) out of 30 in the surgical arm (*p* value for interaction 0.97). Thus, in this highly selected cohort of patients derived from the EVEREST trial, no signals pointing towards a sex-related hazard for the occurrence of short-term adverse events after TEER as compared to surgery was evident.

In the extended 5-year follow-up, initial sex-related discrepancies became apparent. While no differences emerged for the occurrence of the primary endpoint among both sexes (freedom from death, surgery, and recurrent MR grade ≥3 in females: TEER 46.4% vs. surgery 65.0%, *p*-value = 0.15), surgical male patients showed a significant higher freedom from adverse events at follow-up (TEER 42.9% vs. surgery 63.9%, *p* value = 0.03), thus confirming the known prognostic differences evident among sexes in surgical studies. Nonetheless, no evidence for treatment heterogeneity was observed among males and females, suggesting that surgical treatment was associated with better outcomes at follow-up in both sexes [49].

The MITRA-FR trial was an investigator initiated randomized clinical trial aiming to assess whether TEER on top of optimal medical treatment (OMT) was able to confer any prognostic advantage as compared to OMT alone in patients with functional MR [50]. The primary efficacy endpoint was set as the incidence of death or heart failure hospitalizations at 12 months. Female sex was under-represented in this trial as only 75 women (24.4% of the whole study group) were enrolled in the study. The primary endpoint occurred in 16/30 (53.3%) in the TEER group as compared to 25/45 (55.6%) of those allocated to OMT, with no evidence, thus, of significant sex-related discrepancies (OR 0.9; 95% CI 0.4–2.3; *p* value for interaction 0.73). Clearly, these results must be considered in the light of the intrinsic limitations associated with the small sample size of patients enrolled. Currently, no reports derived from the MITRA-FR trial focusing on sex disparities at an extended follow-up are available.

On this topic, additional data derived from the COAPT trial is available. This randomized controlled trial from 2018 aimed at assessing safety and effectiveness of TEER with the MitraClip device in patients with heart failure, reduced ejection failure, and secondary MR who remained symptomatic despite the use of guideline-directed medical therapy [51]. The trial enrolled 614 patients randomized 1:1 to TEER plus OMT vs. OMT alone. The primary endpoint, defined as the composite of death and heart failure hospitalizations at two years, was met, showing a significant reduction in TEER patients as compared to medical therapy. Among study patients, 221 (36%) were female (124 treated with TEER and 97 included in the medical treatment arm). Despite a younger mean age than their male counterparts (mean age 69.5 ± 12.8 vs. 73.8 ± 9.8, *p* < 0.001), women had a higher functional class at enrollment and a lower quality of life as assessed with the Kansas City Cardiomyopathy Questionnaire score. Moreover, female patients were less frequently affected by ischemic cardiomyopathy. Despite comparable procedural success rates among both sexes and a comparable survival benefit conferred by percutaneous treatment, the effect of TEER in reducing heart failure hospitalizations was less pronounced in women compared with men beyond the first year after treatment [52].

### 5.2. Evidence from Registries

Initial registry-derived data reporting sex related differences were published almost 10 years ago. The first available report was derived from a small sample (overall 171 patients of whom 65 were females) assessing patients with predominant functional etiology enrolled in the GRASP registry [53]. This initial report confirmed that, even between TEER patients, women were referred to treatment at an older age than their male counterparts. Authors reported a high success rate in both groups, leading to considerable reduction of MR grade, more pronounced in females. Authors also reported a low rate of procedure-related complications, without significant sex-related differences. Interestingly, while heart failure symptoms were improved early after the intervention (i.e., 30 days) in both groups, a trend toward poorer results in females was evident at 30 days and persisted through 12 months [53].

A subsequent multicenter registry pooling 173 TEER patients, with only 64 women, from Royal Brompton Hospital (London, UK), Rigshospitalet (Copenhagen, Denmark), and Karolinska University Hospital (Stockholm, Sweden) confirmed an older age of referral in women. In line with previous surgical series, even among TEER patients, differences regarding LV volumes were confirmed with females showing smaller LV end systolic and diastolic volumes related to their lower body surface area. Even among this cohort, comparable acute procedural and echocardiographic results after TEER were reported at 12 months between both sexes [54].

The ACCESS EU registry published in 2016 provided a larger comparative analysis, reporting data on 205 women treated with TEER among 14 European sites [55]. Even among patients enrolled in this multicenter registry, females were characterized by an older age at treatment, lower BMI, LV volumes, burden of associated comorbidities, and a higher NYHA class at presentation, thus mirroring surgical experiences. Women were less likely to be treated with multiple clip implantation, this probably due to the smaller annular sizes associated with a smaller body surface area. Nonetheless, comparable safety and efficacy among sexes was confirmed [55].

On this topic, an American perspective is provided by two studies derived from the US National Inpatient Sample. The first study by Doshi et al. assessed the impact of sex disparities in a relatively large analysis derived from administrative data of 521 patients (219 females) treated between 2012 and 2014 [56]. Authors reported no significant differences for in-hospital mortality between both sexes. No differences were also reported for the occurrence of major post-procedural complications such as acute renal failure, stroke, bleeding requiring transfusions, cardiogenic shock, or length of in-hospital stay. Interestingly, cost analysis showed a significantly lower resource allocation per procedure in females (male 50 533 USD vs. female 45 182 USD, *p* = 0.022) [56].

A subsequent analysis was performed querying the National Inpatient Sample database for patients treated by TEER between 2012 and 2016 [57]. Authors reported an exponential increase in the number of procedures performed during the study period among both sexes, leading to 10,015 hospitalizations with 4715 (47.1%) women and 5300 (52.9%) men. Both unmatched and matched analyses (performed after extensive propensity score matching including 26 different variables) showed no differences for the primary outcome of in-hospital mortality between sexes (unmatched cohorts 2.1% in women vs. 2.7% in men, odds ratio [OR] 0.77; 95% confidence interval; 0.48–1.23, *p* = 0.27 matched cohorts 2.4% in women vs. 3.0% in men, OR 0.78; 95% CI: 0.47–1.29, *p* = 0.33) [57]. In the matched cohort, no differences were observed in terms of conversions to surgical mitral valve replacement, cardiac arrest, cardiogenic shock, use of mechanical circulatory support, acute renal insufficiency, need for hemodialysis, respiratory complications, acute myocardial infarction, stroke, complete heart block, cardiac tamponade, discharges to nursing facility, and mean length of hospital stay. These results, even with the intrinsic limitations derived from a retrospective administrative database derived data, confirm the procedural safety of TEER in women also beyond in-hospital mortality.

The German TRAMI registry reported on 828 consecutive patients treated between 2010 and 2013, comparing outcomes among 501 males (60.5%) and 327 females (39.5%) with predominant functional MR without sex-related differences with regard to their etiology [58]. Frailty, reported by the enrolling centers, was much more common in females, justifying a percutaneous approach in 28.9% of all women as compared to 17.4% of men (*p* < 0.001), nonetheless not impacting on procedural success rates. Significantly higher rates of bleedings requiring transfusions and a borderline increase in the rate of vascular complications in women was also reported, highlighting peculiarities of women undergoing TEER [58]. One-year survival was similar among sexes. Nonetheless, while most patients showed a significant clinical benefit in terms of functional capacity and subjective health status 1 year after treatment, females more frequently remained symptomatic after treatment, reporting NYHA classes ≥2 in 45.3% as compared to 31.7% in males (*p* = 0.003).

Recently, sex-related differences in the outcomes after TEER were explored in the EuroSMR registry. A previous analysis derived from the registry confirmed an effective and similar MR reduction with TEER in women and men patients with secondary MR with no sex-related differences in clinical outcomes up to two years of follow-up [59]. More recently, an analysis focusing on the relationship between body surface area and all-cause mortality at two years in patients with functional MR stratified according to sex showed an inverse linear association in males (with lower event rate at higher body surface areas), while a U-shaped relationship in women (with higher event rates at low and high body surface area) [60], partially explaining the commonly perceived higher risk of mortality in females.

Several other percutaneous approaches have been developed such as direct and indirect mitral valve annuloplasty as well as techniques for percutaneous and microinvasive mitral valve replacement or repair [61]. Nonetheless, these approaches are still in their germinal phase and only few data are available allowing sex comparisons [62,63].

In summary, evidence derived from TEER studies mirror surgical experiences showing that females are underrepresented in studies and are often referred to surgery at an older age and at a higher disease stage, suggesting limited access to treatment. Frailty more often justifies a percutaneous approach in women as compared to males. Reassuringly, as a difference from surgical series, no signal towards outcome dissimilarities among sexes is evident both at short- and mid-term follow-up. Nonetheless, persistence of limiting symptoms even after adequate management in women represents an unmet need for treatment.

## 6. Conclusions

**Figure 2:**
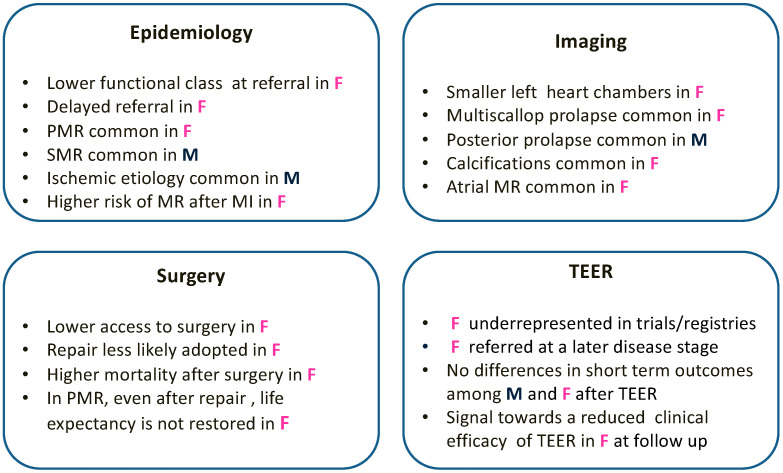
Graphical summary of sex differences in epidemiology, morphology, mechanisms, and treatment of mitral valve regurgitation. MI: myocardial infarction; MR: mitral regurgitation; PMR: primary mitral valve regurgitation; SMR: secondary mitral valve regurgitation; TEER: transcatheter edge-to-edge mitral valve repair.

Sex is associated with relevant differences regarding epidemiology, clinical presentation, and pathological and echocardiographic findings in patients with mitral regurgitation (Figure 2). These differences have an impact on treatment strategies translating into different surgical outcomes among women and men. In patients treated with TEER, sex also represents a relevant factor impacting on therapeutic and interventional strategies. Nonetheless, this does not seem to have a clear impact on short term procedural outcomes. After TEER, the signal suggesting that female sex seems to be associated with a reduced clinical efficacy at follow-up requires further confirmation. After gaining more data from randomized control trials and real-world evidence, there will be a need to evaluate new gender-based recommendation on MR therapy in future guidelines.

## Data Availability

Not applicable.

## References

[1] Nkomo V.T., Gardin J.M., Skelton T.N., Gottdiener J.S., Scott C.G., Enriquez-Sarano M. (2006). Burden of valvular heart diseases: A population-based study. Lancet.

[2] DesJardin J.T., Chikwe J., Hahn R.T., Hung J.W., Delling F.N. (2022). Sex Differences and Similarities in Valvular Heart Disease. Circ. Res..

[3] Otto C.M., Nishimura R.A., Bonow R.O., Carabello B.A., Erwin J.P., Gentile F., Jneid H., Krieger E.V., Mack M., McLeod C. (2021). 2020 ACC/AHA Guideline for the Management of Patients with Valvular Heart Disease: Executive Summary: A Report of the American College of Cardiology/American Heart Association Joint Committee on Clinical Practice Guidelines. Circulation.

[4] Vahanian A., Beyersdorf F., Praz F., Milojevic M., Baldus S., Bauersachs J., Capodanno D., Conradi L., De Bonis M., De Paulis R. (2022). 2021 ESC/EACTS Guidelines for the management of valvular heart disease. Eur. Heart J..

[5] Singh J.P., Evans J.C., Levy D., Larson M.G., Freed L.A., Fuller D.L., Lehman B., Benjamin E.J. (1999). Prevalence and clinical determinants of mitral, tricuspid, and aortic regurgitation (the Framingham Heart Study). Am. J. Cardiol..

[6] Stefano G., Fox K., Schluchter M., Hoit B.D. (2008). Prevalence of Unsuspected and Significant Mitral and Aortic Regurgitation. J. Am. Soc. Echocardiogr..

[7] O’Gara P.T., Mack M.J. (2020). Secondary Mitral Regurgitation. N. Engl. J. Med..

[8] Ruiz J.M.M., Galderisi M., Buonauro A., Badano L., Aruta P., Swaans M.J., Sanchis L., Saraste A., Monaghan M., Theodoropoulos K.C. (2018). Overview of mitral regurgitation in Europe: Results from the European Registry of mitral regurgitation (EuMiClip). Eur. Heart J.-Cardiovasc. Imaging.

[9] Andell P., Li X., Martinsson A., Andersson C., Stagmo M., Zöller B., Sundquist K., Smith J.G. (2017). Epidemiology of valvular heart disease in a Swedish nationwide hospital-based register study. Heart.

[10] Delling F.N., Vasan R.S. (2014). Epidemiology and Pathophysiology of Mitral Valve Prolapse: New Insights into Disease Progression, Genetics, and Molecular Basis. Circulation.

[11] Freed L.A., Levy D., Levine R.A., Larson M.G., Evans J.C., Fuller D.L., Lehman B., Benjamin E.J. (1999). Prevalence and Clinical Outcome of Mitral-Valve Prolapse. N. Engl. J. Med..

[12] Devereux R.B., Brown W.T., Kramer-Fox R., Sachs I. (1982). Inheritance of Mitral Valve Prolapse: Effect of Age and Sex on Gene Expression. Ann. Intern. Med..

[13] Vakamudi S., Jellis C., Mick S., Wu Y., Gillinov A.M., Mihaljevic T., Cosgrove D.M., Svensson L., Cho L. (2018). Sex Differences in the Etiology of Surgical Mitral Valve Disease. Circulation.

[14] Yadgir S., Johnson C.O., Aboyans V., Adebayo O.M., Adedoyin R.A., Afarideh M., Alahdab F., Alashi A., Alipour V., Arabloo J. (2020). Global, Regional, and National Burden of Calcific Aortic Valve and Degenerative Mitral Valve Diseases, 1990–2017. Circulation.

[15] Avierinos J.-F., Inamo J., Grigioni F., Gersh B., Shub C., Enriquez-Sarano M. (2008). Sex Differences in Morphology and Outcomes of Mitral Valve Prolapse. Ann. Intern. Med..

[16] Nishimura R.A., McGoon M.D., Shub C., Miller F.A., Ilstrup D.M., Tajik A. (1985). Echocardiographically Documented Mitral-Valve Prolapse. Long-term follow-up of 237 patients. N. Engl. J. Med..

[17] Putnam A.J., Kebed K., Mor-Avi V., Rashedi N., Sun D., Patel B., Balkhy H., Lang R.M., Patel A.R. (2020). Prevalence of mitral annular disjunction in patients with mitral valve prolapse and severe regurgitation. Int. J. Cardiovasc. Imaging.

[18] Sriram C.S., Syed F.F., Ferguson M.E., Johnson J.N., Enriquez-Sarano M., Cetta F., Cannon B.C., Asirvatham S.J., Ackerman M.J. (2013). Malignant Bileaflet Mitral Valve Prolapse Syndrome in Patients with Otherwise Idiopathic Out-of-Hospital Cardiac Arrest. J. Am. Coll. Cardiol..

[19] Groeneveld S.A., Kirkels F.P., Cramer M.J., Evertz R., Haugaa K.H., Postema P.G., Prakken N.H.J., Teske A.J., Wilde A.A.M., Velthuis B.K. (2022). Prevalence of Mitral Annulus Disjunction and Mitral Valve Prolapse in Patients with Idiopathic Ventricular Fibrillation. J. Am. Heart Assoc..

[20] Namazi F., Bijl P., Vo N.M., Wijngaarden S.E., Marsan N.A., Delgado V., Bax J.J. (2021). Sex differences in prognosis of significant secondary mitral regurgitation. ESC Heart Fail..

[21] Stolfo D., Uijl A., Vedin O., Strömberg A., Foxen U.L., Rosano G.M., Sinagra G., Dahlström U., Savarese G. (2019). Sex-Based Differences in Heart Failure Across the Ejection Fraction Spectrum. JACC Heart Fail..

[22] Ho J.E., Brouwers F.P., Enserro D., Shah S.J., Psaty B.M., Bartz T.M., Santhanakrishnan R., Lee D.S., Liu K., Blaha M.J. (2016). Abstract 11958: Predicting Heart Failure with Preserved and Reduced Ejection Fraction: The International Collaboration on Heart Failure Subtypes. Circ. Heart Fail..

[23] Fleury M.-A., Clavel M.-A. (2021). Sex and Race Differences in the Pathophysiology, Diagnosis, Treatment, and Outcomes of Valvular Heart Diseases. Can. J. Cardiol..

[24] Giustino G., Overbey J., Taylor D., Ailawadi G., Kirkwood K., DeRose J., Gillinov M.A., Dagenais F., Mayer M.-L., Moskowitz A. (2019). Sex-Based Differences in Outcomes after Mitral Valve Surgery for Severe Ischemic Mitral Regurgitation. JACC Heart Fail..

[25] Coffey S., Roberts-Thomson R., Brown A., Carapetis J., Chen M., Enriquez-Sarano M., Zühlke L., Prendergast B.D. (2021). Global epidemiology of valvular heart disease. Nat. Rev. Cardiol..

[26] Nitsche C., Koschutnik M., Kammerlander A., Hengstenberg C., Mascherbauer J. (2020). Gender-specific differences in valvular heart disease. Wien. Klin. Wochenschr..

[27] Patlolla S.H., Schaff H.V., Nishimura R.A., Geske J.B., Lahr B.D., Lee A.T., Eleid M.F., Ommen S.R., Dearani J.A. (2021). Mitral Annular Calcification in Obstructive Hypertrophic Cardiomyopathy: Prevalence and Outcomes. Ann. Thorac. Surg..

[28] Deferm S., Bertrand P.B., Verbrugge F.H., Verhaert D., Rega F., Thomas J.D., Vandervoort P.M. (2019). Atrial Functional Mitral Regurgitation. J. Am. Coll. Cardiol..

[29] Gual-Capllonch F., de Ibarra J.I.S., Bayés-Genís A., Delgado V. (2022). Atrial Mitral and Tricuspid Regurgitation: Sex Matters. A Call for Action to Unravel the Differences Between Women and Men. Front. Cardiovasc. Med..

[30] Bach D.S., Radeva J.I., Birnbaum H.G., Fournier A.A., Tuttle E.G. (2007). Prevalence, referral patterns, testing, and surgery in aortic valve disease: Leaving women and elderly patients behind?. J. Heart Valve Dis..

[31] Wang A., Grayburn P., Foster J.A., McCulloch M.L., Badhwar V., Gammie J.S., Costa S.P., Benitez R.M., Rinaldi M.J., Thourani V.H. (2016). Practice gaps in the care of mitral valve regurgitation: Insights from the American College of Cardiology mitral regurgitation gap analysis and advisory panel. Am. Heart J..

[32] Mantovani F., Clavel M.A., Michelena H.I., Suri R.M., Schaff H.V., Enriquez-Sarano M. (2016). Comprehensive Imaging in Women with Organic Mitral Regurgitation: Implications for Clinical Outcome. JACC Cardiovasc. Imaging.

[33] Yi K., Gao J., Wang W.-X., Ma Y.-H., Wang W., He S.E., Xu X.-M., Li P.-F., You T. (2023). Gender-related differences on outcome following transcatheter mitral valve repair (TMVR): A systematic review and meta-analysis. J. Cardiothorac. Surg..

[34] Goebel B., Heck R., Hamadanchi A., Otto S., Doenst T., Jung C., Lauten A., Figulla H.R., Schulze P.C., Poerner T.C. (2018). Vena contracta area for severity grading in functional and degenerative mitral regurgitation: A transoesophageal 3D colour Doppler analysis in 500 patients. Eur. Heart J.-Cardiovasc. Imaging.

[35] Brady B., King G., Murphy R.T., Walsh D. (2022). Myocardial strain: A clinical review. Ir. J. Med. Sci..

[36] Ueyama H., Kuno T., Takagi H., Krishnamoorthy P., Prandi F.R., Palazzuoli A., Sharma S.K., Kini A., Lerakis S. (2022). Prognostic value of left ventricular global longitudinal strain in mitral regurgitation: A systematic review. Heart Fail. Rev..

[37] Bsc A.C.M.D., Afoke J., Punjabi P.P., Kanaganayagam G.S. (2021). Global longitudinal strain to determine optimal timing for surgery in primary mitral regurgitation: A systematic review. J. Card. Surg..

[38] Uretsky S., Animashaun I.B., Sakul S., Aldaia L., Marcoff L., Koulogiannis K., Argulian E., Rosenthal M., Wolff S.D., Gillam L.D. (2022). American Society of Echocardiography Algorithm for Degenerative Mitral Regurgitation: Comparison with CMR. JACC Cardiovasc. Imaging.

[39] Vermes E., Iacuzio L., Levy F., Bohbot Y., Renard C., Gerber B., Maréchaux S., Tribouilloy C. (2022). Role of Cardiovascular Magnetic Resonance in Native Valvular Regurgitation: A Comprehensive Review of Protocols, Grading of Severity, and Prediction of Valve Surgery. Front. Cardiovasc. Med..

[40] Badiani S., van Zalen J., Althunayyan A., Al-Borikan S., Treibel T., Marshall A., Patel N., Bhattacharyya S., Lloyd G. (2021). Natriuretic peptide release during exercise in patients with valvular heart disease: A systematic review. Int. J. Clin. Pract..

[41] Gallo G., Forte M., Stanzione R., Cotugno M., Bianchi F., Marchitti S., Berni A., Volpe M., Rubattu S. (2020). Functional Role of Natriuretic Peptides in Risk Assessment and Prognosis of Patients with Mitral Regurgitation. J. Clin. Med..

[42] Detaint D., Messika-Zeitoun D., Avierinos J.-F. (2005). B-Type Natriuretic Peptide in Organic Mitral Regurgitation: Determinants and Impact on Outcome. Circulation.

[43] Redfield M.M., Rodeheffer R.J., Jacobsen S.J., Mahoney D.W., Bailey K.R., Burnett J.C. (2002). Plasma brain natriuretic peptide concentration: Impact of age and gender. J. Am. Coll. Cardiol..

[44] Cediel G., Codina P., Spitaleri G., Domingo M., Santiago-Vacas E., Lupón J., Bayes-Genis A. (2021). Gender-Related Differences in Heart Failure Biomarkers. Front. Cardiovasc. Med..

[45] Ibrahim M.F., Paparella D., Ivanov J., Buchanan M.R., Brister S.J. (2003). Gender-related differences in morbidity and mortality during combined valve and coronary surgery. J. Thorac. Cardiovasc. Surg..

[46] Vassileva C.M., McNeely C., Mishkel G., Boley T., Markwell S., Hazelrigg S. (2013). Gender Differences in Long-Term Survival of Medicare Beneficiaries Undergoing Mitral Valve Operations. Ann. Thorac. Surg..

[47] Nashef S.A., Roques F., Sharples L.D., Nilsson J., Smith C., Goldstone A.R., Lockowandt U. (2012). EuroSCORE II. Eur. J. Cardiothorac. Surg..

[48] Feldman T., Foster E., Glower D.D., Kar S., Rinaldi M.J., Fail P.S., Smalling R.W., Siegel R., Rose G.A., Engeron E. (2011). Percutaneous repair or surgery for mitral regurgitation. N. Engl. J. Med..

[49] Feldman T., Kar S., Elmariah S., Smart S.C., Trento A., Siegel R.J., Apruzzese P., Fail P., Rinaldi M.J., Smalling R.W. (2015). Randomized Comparison of Percutaneous Repair and Surgery for Mitral Regurgitation: 5-Year Results of EVEREST II. J. Am. Coll. Cardiol..

[50] Obadia J.-F., Messika-Zeitoun D., Leurent G., Iung B., Bonnet G., Piriou N., Lefèvre T., Piot C., Rouleau F., Carrié D. (2018). Percutaneous Repair or Medical Treatment for Secondary Mitral Regurgitation. N. Engl. J. Med..

[51] Stone G.W., Lindenfeld J., Abraham W.T., Kar S., Lim D.S., Mishell J.M., Whisenant B., Grayburn P.A., Rinaldi M., Kapadia S.R. (2018). Transcatheter Mitral-Valve Repair in Patients with Heart Failure. N. Engl. J. Med..

[52] Kosmidou I., Lindenfeld J., Abraham W.T., Rinaldi M.J., Kapadia S.R., Rajagopal V., Sarembock I.J., Brieke A., Gaba P., Rogers J.H. (2021). Sex-Specific Outcomes of Transcatheter Mitral-Valve Repair and Medical Therapy for Mitral Regurgitation in Heart Failure. JACC Heart Fail..

[53] Attizzani G.F., Ohno Y., Capodanno D., Cannata S., Dipasqua F., Immé S., Mangiafico S., Barbanti M., Ministeri M., Cageggi A. (2015). Gender-related clinical and echocardiographic outcomes at 30-day and 12-month follow up after MitraClip implantation in the GRASP registry: Gender-Related Outcomes after MitraClip. Cathet. Cardiovasc. Intervent..

[54] Estévez-Loureiro R., Settergren M., Winter R., Jacobsen P., Dall’Ara G., Sondergaard L., Cheung G., Pighi M., Ghione M., Ihlemann N. (2015). Effect of Gender on Results of Percutaneous Edge-to-Edge Mitral Valve Repair with MitraClip System. Am. J. Cardiol..

[55] Gafoor S., Sievert H., Maisano F., Baldus S., Schaefer U., Hausleiter J., Butter C., Ussia G.P., Geist V., Widder J.D. (2016). Gender in the ACCESS-EU registry: A prospective, multicentre, non-randomised post-market approval study of MitraClip^®^ therapy in Europe. EuroIntervention.

[56] Doshi R., Shlofmitz E., Vadher A., Shah J., Meraj P. (2018). Impact of sex on short term in-hospital outcomes with transcatheter edge-to-edge mitral valve repair. Cardiovasc. Revasc. Med..

[57] Elbadawi A., Elzeneini M., Thakker R., Mahmoud K., Elgendy I.Y., Megaly M., Hamed M., Omer M.A., Chowdhury M., Ogunbayo G. (2020). Sex Differences in In-Hospital Outcomes of Transcatheter Mitral Valve Repair (from a National Database). Am. J. Cardiol..

[58] Werner N., Puls M., Baldus S., Lubos E., Bekeredjian R., Sievert H., Schofer J., Kuck K.-H., Möllmann H., Hehrlein C. (2020). Gender-related differences in patients undergoing transcatheter mitral valve interventions in clinical practice: 1-year results from the German TRAMI registry. Catheter. Cardiovasc. Interv..

[59] Park S.-D., Orban M., Karam N., Lubos E., Kalbacher D., Braun D., Stolz L., Neuss M., Butter C., Praz F. (2021). Sex-Related Clinical Characteristics and Outcomes of Patients Undergoing Transcatheter Edge-to-Edge Repair for Secondary Mitral Regurgitation. JACC Cardiovasc. Interv..

[60] Higuchi S., Orban M., Adamo M., Giannini C., Melica B., Karam N., Praz F., Kalbacher D., Lubos E., Stolz L. (2023). Sex-specific impact of anthropometric parameters on outcomes after transcatheter edge-to-edge repair for secondary mitral regurgitation. Int. J. Cardiol..

[61] D’onofrio A., Gerosa G. (2015). WITHDRAWN: Shifting A Paradigm of Cardiac Surgery: From Minimally Invasive To Micro-Invasive. J. Thorac. Cardiovasc. Surg..

[62] Vairo A., Gaiero L., Marro M., Russo C., Bolognesi M., Soro P., Gallone G., Fioravanti F., Desalvo P., D’ascenzo F. (2023). New Echocardiographic Parameters Predicting Successful Trans-Ventricular Beating-Heart Mitral Valve Repair with Neochordae at 3 Years: Monocentric Retrospective Study. J. Clin. Med..

[63] Wild M.G., Kreidel F., Hell M.M., Praz F., Mach M., Adam M., Reineke D., Ruge H., Ludwig S., Conradi L. (2022). Transapical mitral valve implantation for treatment of symptomatic mitral valve disease: A real-world multicentre experience. Eur. J. Heart Fail..

